# Enhanced Expression of Pullulanase in *Bacillus subtilis* by New Strong Promoters Mined From Transcriptome Data, Both Alone and in Combination

**DOI:** 10.3389/fmicb.2018.02635

**Published:** 2018-11-02

**Authors:** Fanqiang Meng, Xiaoyu Zhu, Ting Nie, Fengxia Lu, Xiaomei Bie, Yingjian Lu, Frances Trouth, Zhaoxin Lu

**Affiliations:** ^1^College of Food Science and Technology, Nanjing Agricultural University, Nanjing, China; ^2^Department of Food Science and Nutrition, University of Maryland, College Park, MD, United States; ^3^Department of Plant Science and Landscape Architecture, University of Maryland, College Park, MD, United States

**Keywords:** *Bacillus*, transcriptome, pullulanase, multi-promoter, fermentation

## Abstract

Pullulanase plays an important role as a starch hydrolysis enzyme in the production of bio-fuels and animal feed, and in the food industry. Compared to the methods currently used for pullulanase production, synthesis by *Bacillus subtilis* would be safer and easier. However, the current yield of pullulanase from *B. subtilis* is low to meet industrial requirements. Therefore, it is necessary to improve the yield of pullulanase by *B. subtilis*. In this study, we mined 10 highly active promoters from *B. subtilis* based on transcriptome and bioinformatic data. Individual promoters and combinations of promoters were used to improve the yield of pullulanase in *B. subtilis* BS001. Four recombinant strains with new promoters (Phag, PtufA, PsodA, and PfusA) had higher enzyme activity than the control (PamyE). The strain containing PsodA+fusA (163 U/mL) and the strain containing PsodA+fusA+amyE (336 U/mL) had the highest activity among the analyzed dual- and triple-promoter construct stains in shake flask, which were 2.29 and 4.73 times higher than that of the strain with PamyE, respectively. Moreover, the activity of the strain containing PsodA+fusA+amyE showed a maximum activity of 1,555 U/mL, which was 21.9 times higher than that of the flask-grown PamyE strain in a 50-liter fermenter. Our work showed that these four strong promoters mined from transcriptome data and their combinations could reliably increase the yield of pullulanase in quantities suitable for industrial applications.

## Introduction

Industrial starch fermentation for the production of alcohols, amino acids, nucleotides, antibiotics, and high-glucose and high-maltose syrups (Ram and Venkatasubramanian, 1982; Malviya et al., 2010) relies on pullulanase to degrade α-1,6-glycosidic bonds to improve the efficiency of starch hydrolysis (Reddy et al., 2015). Pullulanase is also used to produce high-amylose starch, resistant starch, slow-digestion starch, maltooligosaccharides, and branched cyclodextrins (Shikaishi et al., 2014; Li et al., 2017).

Because pullulanase is used in these applications, a safe, low-cost, high-yield production method is needed. Although heterologous expression of pullulanase in *Escherichia coli* under various conditions has yielded as much as 580 U/mL (Nie et al., 2013) and 2523.5 U/mL (Zou et al., 2014), there are many restrictions for its use in foods, feeds, and pharmaceuticals because of the endotoxins and exotoxins produced by *E. coli*. Safer alternative species have been used for pullulanase production, including *Bacillus subtilis* (24.5 U/mL) (Song et al., 2016) and *Pichia pastoris* (350 U/mL) (Xu et al., 2006); however, the yields from these strains are relatively low. To solve these problems, researchers have isolated new types of pullulanase enzymes from various microorganisms, such as *Bacillus* sp. AV-7 (Kunamneni and Singh, 2006), *Thermus thermophiles* (Wu et al., 2014), *Bacillus deramificans* (Duan et al., 2013), *Anoxybacillus* sp. SK3-4 (Kahar et al., 2016), and *Bacillus naganoensis*. In addition, the yield of pullulanase has been increased through mutation breeding (2.82 U/ml) (Wang et al., 2014), protein engineering (46.9 U/ml) (Chen et al., 2016; Nisha and Satyanarayana, 2016), and the manipulation of culture condition (543 U/ml) (Zou et al., 2016). Despite these efforts, the yield of pullulanase from these strains is still too low to meet industrial demand.

*B. subtilis* is a viable species for improving pullulanase yield because it is a generally recognized as safe (GRAS) microbial-derived product (Ming et al., 2010). Therefore, we chose to use *B. subtilis* for our pullulanase production study. Protein yield is known to be closely related to the strength of the promoter; thus, a strong promoter is a necessary requirement for high protein yield (Blazeck et al., 2012). The most well-known promoter in *B. subtilis* is the cytidine deaminase (*ccd*) promoter P43 (Wu et al., 1991), which has been used to express GFP (Kong et al., 2009), β-galactosidase, staphylokinase (Kim et al., 2008) and alkaline protease (Kim et al., 2008). Yang et al. isolated a strong *B. subtilis* promoter (Plaps) that is 13 times stronger than the P43 promoter by using a promoter trapping system. Inducible promoters have also been widely used in *B. subtilis*; including promoters that are regulated by xylose, sucrose (Biedendieck et al., 2007), maltose (Biedendieck et al., 2007; Yue et al., 2017), starch, phosphates (Abdel-Fattah et al., 2005; Makarewicz et al., 2006), citric acid (Yamamoto et al., 2000), tetracycline (Geissendörfer and Hillen, 1990), and glycine (Phan and Schumann, 2007).

Moreover, promoters an also be combined to form a multiple-promoter complexes to further enhance the expression (Zhang et al., 2017), and have been shown to increase enzyme production 1.6- (Yang et al., 2013), and 12-fold (Kang et al., 2010). Zhang et al. (2017) designed a dual-promoter expression system, PhpaII-PamyQ. Using this system, they increased enzyme activity to 571.2 U/mL in a 3 L fermenter, which was 18.7 times the activity obtained in shake flasks. Guan C. R. et al. (2016) showed that amino peptidase could be expressed in *B. subtilis* by the synthetic dual promoter PgsiB-PHpaII. Using this system, the obtained enzyme activity was 88.86 U/mL in shake flasks and 205 U/mL in a 5 L fermenter. In addition, the core elements of promoters, including the −35 and −10 regions (Jiao et al., 2017) and ribosome recognition site (Wang and Doi, 1984), have been optimized to enhance the promoter strength.

One bioinformatic method for selecting candidate strong promoters to improve production efficiency is analyzing the amount of mRNA expressed in a transcriptome, which should represent the strength of the promoter (McCleary, 2009). This method has been used for other applications to increase production efficiency, thus saving time and reducing costs. For example, Liu et al. (2017) analyzed the top 10 most highly expressed genes and operons among 3,959 genes and 1,249 operons in transcriptome data from *Bacillus licheniformis* ATCC14580. Using this method, a novel high-efficiency promoter (PBL9) was identified, which showed 23% higher expression than P43 in *B. subtilis*. Geng et al. (2014) cloned a root-specific promoter and developed a high-yield screening system in peanut by establishing a simple digital expression profile based on Illumina sequencing data from peanut. However, no study has utilized transcriptome data to select highly active promoters in *B. subtilis* based on gene expression levels.

The major objective of this study was to improve the yield of pullulanase production by *B. subtilis* using different promoters. To this end, we first chose three *B. subtilis* transcriptome data sets to screen for strong promoters. Next, pullulanase expression driven by the selected promoters was evaluated in *B. subtilis* BS001. Then, these promoters were combined to generate dual- or triple-promoter expression systems to improve yield.

## Materials and methods

### Microbial strains and vectors

The bacterial strains used in this study are described in Table 1. *E. coli* was cultured in LB broth at 37°C*. B. subtilis* was cultured in CSA medium (maltose, 40 g/L; cotton seed powder, 10 g/L; soybean meal, 10 g/L; ammonium sulfate, 5 g/L; ammonium citrate, 10 g/L; dipotassium hydrogen phosphate, 9 g/L; magnesium sulfate, 0.2 g/L; manganese sulfate, 0.05 g/L; ferrous sulfate, 0.05 g/L; and calcium chloride, 1 g/L, which was adjusted to pH 6.0 before sterilization at 121°C for 20 min, pH 5.8 after sterilization) at 37°C.

**Table 1:** Strains used in this study.

**Strains and vectors**	**Description**	**Application**	**Source**
*E.coli* DH5α	F-,SupE44ΔlacU169(ϕ80lacZΔM15) hsdR17 recA1 endA1 gyrA96 thi-1 relA1	Plasmid sub cloning	Vazyme.Ltd
*Bacillus subtilis* BS001	*Bacillus subtilis* 168 derivative, The following genes have been deleted: *aprE, nprE, uvrX, gudB* and *tuaA*.	Expression host	Lab stock
pCBS	*Bacillus* thermo-sensitive recombinant vector	Recombinant vector	Lab stock
pCBS1	pCBS with *pulA* gene	Recombinant vector	this study
pCBS2	pCBS with *pulA* and signal peptide amyE	Recombinant vector	this study
pCBS3	pCBS with *pulA*, SPamyE, and mRNA stable sequence	Recombinant vector	this study
pCBS4	pCBS3 with PamyE	Recombinant vector	this study
pCBS5	pCBS3 with Phag	Recombinant vector	this study
pCBS6	pCBS3 with PtufA	Recombinant vector	this study
pCBS7	pCBS3 with PcspD	Recombinant vector	this study
pCBS8	pCBS3 with PyqeY	Recombinant vector	this study
pCBS9	pCBS3 with PsodA	Recombinant vector	this study
pCBS10	pCBS3 with PfusA	Recombinant vector	this study
pCBS11	pCBS3 with PgapA	Recombinant vector	this study
pCBS12	pCBS3 with PahpF	Recombinant vector	this study
pCBS13	pCBS3 with PglnA	Recombinant vector	this study
pCBS14	pCBS3 with Pmdh	Recombinant vector	this study
pCBS15	pCBS3 with PsodA+hag	Recombinant vector	this study
pCBS16	pCBS3 with PsodA+tufA	Recombinant vector	this study
pCBS17	pCBS3 with PdosA+fusA	Recombinant vector	this study
pCBS18	pCBS3 with PsodA+amyE	Recombinant vector	this study
pCBS19	pCBS3 with Phag+tufA	Recombinant vector	this study
pCBS20	pCBS3 with Phag+fusA	Recombinant vector	this study
pCBS21	pCBS3 with Phag+amyE	Recombinant vector	this study
pCBS22	pCBS3 with PtufA+fusA	Recombinant vector	this study
pCBS23	pCBS3 with PtufA+amyE	Recombinant vector	this study
pCBS24	pCBS3 with PfusA+amyE	Recombinant vector	this study
pCBS25	pCBS3 with Phag+hag	Recombinant vector	this study
pCBS26	pCBS3 with PtufA+tufA	Recombinant vector	this study
pCBS27	pCBS3 with PsodA+sodA	Recombinant vector	this study
pCBS28	pCBS3 with PfusA+fusA	Recombinant vector	this study
pCBS29	pCBS3 with PamyE+amyE	Recombinant vector	this study
pCBS30	pCBS3 with PsodA+hag+tufA	Recombinant vector	this study
pCBS31	pCBS3 with PsodA+hag+fusA	Recombinant vector	this study
pCBS32	pCBS3 with PsodA+hag+amyE	Recombinant vector	this study
pCBS33	pCBS3 with PsodA+tufA+fusA	Recombinant vector	this study
pCBS34	pCBS3 with PsodA+tufA+amyE	Recombinant vector	this study
pCBS35	pCBS3 with PsodA+fusA+amyE	Recombinant vector	this study
pCBS36	pCBS3 with Phag+tufA+fusA	Recombinant vector	this study
pCBS37	pCBS3 with Phag+tufA+amyE	Recombinant vector	this study
pCBS38	pCBS3 with Phag+fusA+amyE	Recombinant vector	this study
pCBS39	pCBS3 with PtufA+fusA+amyE	Recombinant vector	this study
pCBS40	pCBS3 with PsodA+sodA+sodA	Recombinant vector	this study
pCBS41	pCBS3 with Phag+hag+hag	Recombinant vector	this study
pCBS42	pCBS3 with PtufA+tufA+tufA	Recombinant vector	this study
pCBS43	pCBS3 with PamyE+amyE+amyE	Recombinant vector	this study
pCBS44	pCBS3 with PfusA+fusA+fusA	Recombinant vector	this study

The vectors used in this study are also listed in Table 1. The gene ID and position of all promoters are shown in Table 2. The sequence of pulA and promoters were shown in Supplementary Material. Super Pfu DNA polymerase, DNA markers, restriction endonucleases, reverse transcriptase, and TRIzol reagent (for RNA extraction) were purchased from TaKaRa Biotechnology (Dalian, China). The pullulanase gene (*pulA*) and primers (Table S1) were synthesized by Genscript (Nanjing, China).

**Table 2:** the sequence of pulA, promoters and signal peptides.

**Gene**	**Origin**	**Description**	**Accession number**	**Position**
amyE-up	*B. subtilis*	Amylase gene	NC_000964.3	327348-327936
amyE-down	*B. subtilis*			328748-329395
mRNA stable sequence	*B. thuringiensis*	Shine-Dalgarno mRNA stabilizing sequence of *cry*IIIA	AJ237900.1	450-552
*pulA*	*B. acidpullulyticus*	Artificially synthesized codon-optimized gene	MH411123	1-2478
PamyE	*B. subtilis*	Amylase	NC_000964.3	327268-327415
Phag	*B. subtilis*	Flagellin	NC_000964.3	3635836-3635693
PtufA	*B. subtilis*	elongation factor Tu	NC_000964.3	132761-132879
PcspD	*B. subtilis*	cold shock protein	NC_000964.3	2307664-2307904
PyqeY	*B. subtilis*	hypothetical protein	NC_000964.3	2620520-2620357
PsodA	*B.subtilis*	superoxide dismutase	NC_000964.3	2586043-2586220
PfusA	*B. subtilis*	elongation factor G	NC_000964.3	130551-130683
PgapA	*B. subtilis*	glyceraldehyde-3-phosphate dehydrogenase 1	NC_000964.3	3482706-3482835
PahpF	*B. subtilis*	alkyl hydroperoxide reductase	NC_000964.3	4118851-4118949
PglnA	*B. subtilis*	glutamine synthetase	NC_000964.3	1877850-1877958
Pmdh	*B. subtilis*	malate dehydrogenase	NC_000964.3	2979673-2979770
SPamyE	*B. subtilis*	Signal peptide of amylase	NC_000964.3	327618-327716

### Analysis of transcriptome data

Target gene yield is closely related to the strength of the promoter in the host strain. To select a strong promoter, three *B. subtilis* subsp. subtilis str. 168 transcriptome data sets were downloaded from the NCBI SRA database. The transcriptome accession numbers are ERR1223408 (https://trace.ncbi.nlm.nih.gov/Traces/sra/?run=ERR1223408), SRR3488633 (https://trace.ncbi.nlm.nih.gov/Traces/sra/?run=SRR3488633), and SRR3466199 (https://trace.ncbi.nlm.nih.gov/Traces/sra/?run=SRR3466199), which were used as controls in the respective studies (described in Table 3). The sequence data were processed by using the NGS QC Toolkit (2.3.3) to remove low-quality reads. The *B. subtilis* 168 genome (NC_000964.3) was used as a reference for transcript identification by Bowtie 2 (Version 2.2.9). Gene expression levels were analyzed by RPKM (Reads Per Kilo-bases per Million-reads), a standard method for the analysis of gene expression levels, in the HTSeq software package (Version 0.6.1). Functional annotation of the genes was based on databases, e.g., http://bacteria.ensembl.org/Bacillus_subtilis_subsp_subtilis_str_168/Info/Index, http://networks.systemsbiology.net/bsu, and http://genome2d.molgenrug.nl/. Then, the top 200 most highly expressed genes in each transcriptome were selected and analyzed. Genes present in all three sets were sorted by RPKM value. Finally, the promoters of these genes were predicted by Promoter Scan software (https://www.ncbi.nlm.nih.gov/Class/NAWBIS/Modules/DNA/dna21b.html), BPROM (http://www.softberry.com/berry.phtml?topic=bprom&group=programs&subgroup=gfindb), and BDGP (http://www.fruitfly.org/seq_tools/promoter.html) to identify the ribosome binding sites, transcription initiation sites, and TATA boxes.

**Table 3:** the information of three transcriptomes of *B. subtilis* 168.

**Accession number**	**Purpose**	**Description**	**References**
ERR1223408	*B. subtilis* expression after infection with the virus f29	No-infect, control group. LB medium with 5 mM MgSO_4_ at 37°C.	Mojardín and Salas, 2016
SRR3488633	*B. subtilis* spore outgrowth in high-salinity environments	No-salt, control group. Spizizen minimal medium at 37°C.	Nagler et al., 2016
SRR3466199	*B. subtilis* expression treated by mitomycin	No-mitomycin, control group. MMB medium at 37°C.	Forrest et al., 2017

### Design of the expression box

**Figure 1:**
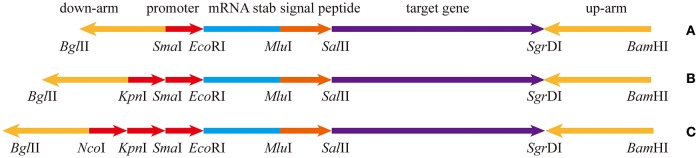
The expression box of pullulanase containing different number of promoters. **(A)** The expression box with single promoter. **(B)** The expression box with dual-promoter. **(C)** The expression box with triple-promoter. The upstream and downstream homology arms are upstream and downstream of *amyE*; mRNA stabilizing sequence is from Shine-Dalgarno mRNA stabilizing sequence of *cry*IIIA of *B. thuringiensis*; the signal peptide is from amyE of *B. subtilis* 168. The target gene is artificially synthesized codon-optimized pullulanase gene (pulA) based on the pullulanase gene of *B. acidpullulyticus*.

The pullulanase expression unit in *B subtilis* can be divided into four parts, the promoter, the Shine-Dalgarno mRNA stabilizing sequence (STAB-SD), the signal peptide sequence, and the pullulanase gene (Figure 1). Several promoters, including Phag, PtufA, PcapD, PyqeY, PsodA, PfusA, PgapA, PahpF, PglnA, and Pmdh, were mined from the analyzed transcriptome data. The *amyE* promoter (PamyE) was used as a control promoter. The STAB-SD of *cry*3A from *Bacillus thuringiensis* was selected for use in the pullulanase expression system, as we hoped that this sequence could improve the stability of the mRNA and increase the yield of target gene (Park et al., 1999). The signal peptide was from *B. subtilis* 168 *amyE*. The reference pullulanase sequence was a type I pullulanase from *Bacillus acidpullulyticus* (Accession number: 2WAN_A, GI: 229597615). The codons of the gene were optimized for expression in *B. subtilis* based on the codon preference of *B. subtilis* 168 (GenBank accession number MH411123) by using codon optimization software (http://www.jcat.de/). Selected promoters were also combined into dual and/or triple promoter systems to increase expression.

### Pullulanase expressed with different promoters and combinations of promoters

The homologous arms of the amylase gene (*amyE*) and the *pulA* expression box were ligated to the pCBS expression vector according to general methods (Sambrook and Russell, 2006). Then, the expression vectors were transformed into *B. subtilis* BS001 according to the method described by Dubnau (Gryczan et al., 1978). Recombinant strains were selected by resistance to erythromycin. Positive transformants were selected by blue-white screening after incubation at 45°C for 12 h. All engineered strains were cultured in CSA medium at 37°C and 180 rpm for 48 h. Extracellular enzyme activity was measured according to the method of Kahar et al. (2016), and the proteins in the supernatant were separated by SDS-PAGE according to “The Condensed Protocols from Molecular Cloning: a Laboratory Manual” (Sambrook and Russell, 2006). Protein content was determined by the Coomassie Brilliant Blue method. Specific enzyme activity (U/mg) was the enzyme activity (U/mL) divided by the protein content (mg/mL).

### Detection of promoter activity by qPCR

All engineered strains were cultured in flasks at 37°C for 24 and 48 h. Then, samples removed were centrifuged at 12,000 × g for 5 min, and the RNA was extracted by using TRIzol according to a previously described method (Sambrook and Russell, 2006). RNA was reverse transcribed into cDNA, and pullulanase expression was detected by qPCR and the ΔΔCT method. In this study, the reference gene was the 16S ribosome gene.

### Pullulanase yield from engineered strains in shake flasks and 50-L fermenters

Engineered strains containing pullulanase under PamyE, PsodA+fusA, and PsodA+fusA+amyE were cultured in 250 mL flasks containing 50 mL of CSA medium (pH 5.8) at 37°C for 48 h. Extracellular enzyme activity was determined every 4 h. The strain containing PsodA+fusA+amyE was cultured in 1,000 mL flasks containing 200 mL of CSA medium at 37°C for ~12 h until the cell density (OD_600_) reached 20. Then, the cells were transferred to a 50 L fermenter (10% inoculum). The fermentation was conducted for 48 h under the following conditions: the total sugar content was maintained at 0.5–1.0% by adding 50% maltose syrup, dissolved oxygen was maintained at >20% by controlling stirring speed and ventilation, the pH was maintained at 5.8 or 6.5 by adding ammonia water, and the temperature was maintained at 37 or 33°C. Extracellular enzyme activity was determined every 4 h.

The pullulanase yield in 50-L fermenters was shown one experiment data in section The Pullulanase Yield From Engineered Strains in Flasks and 50-L Fermenters. The other experiments were repeated four times, and the data were analyzed by one-way analysis of variance (ANOVA) with Tukey's multiple comparison tests for *post-hoc* comparisons in SPSS (version 17.0). A *p*-value < 0.05 was considered statistically significant.

## Results

### Selection of strong promoters based on transcriptome data

**Figure 2:**
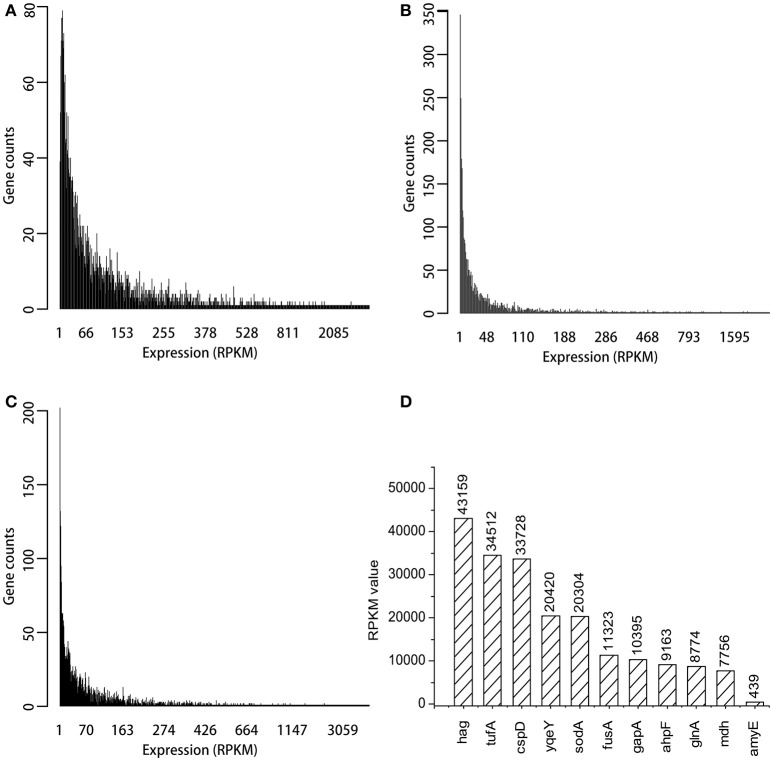
Expression distribution of all genes in three transcriptomes and RPKM values of top ten genes. **(A)** The expression distribution in ERR1223408. **(B)** The expression distribution in SRR3488633. **(C)** The expression distribution in SRR3466199. **(D)** The top 10 expressed genes and amyE based on RPKM values.

Three transcriptome data sets from *B. subtilis*, ERR1223408, SRR3488633, and SRR3466199 were analyzed by using bioinformatic methods. The genome of *B. subtilis* 168 was used to annotate the transcriptomes, and 4,217 genes were identified. The expression levels of most genes was low, and the RPKM values were <200 as shown in Figures 2A–C. The top 200 RPKM value of genes were identified in each of the three transcriptomes data sets, and within this group of genes, there were 105 that were present in all three transcriptomes. The RPKM value of the 10 most highly expressed genes that were represented in all three transcriptomes and *amyE* are shown in Figure 2D. The *hag* gene (flagellin) had the highest RPKM value (43159), which was 98 times that of *amyE* (439). The RPKM value of *tufA* gene (elongation factor Tu) and *cspD* gene (cold shock protein) were >30,000. The promoter regions of the selected genes was predicted by Promoter Scan, BPROM, and BDGP as described in Table 2, and the sequences were shown in Supplementary Material.

### The effect of different promoters and their combinations on the mRNA of pullulanase

**Figure 3:**
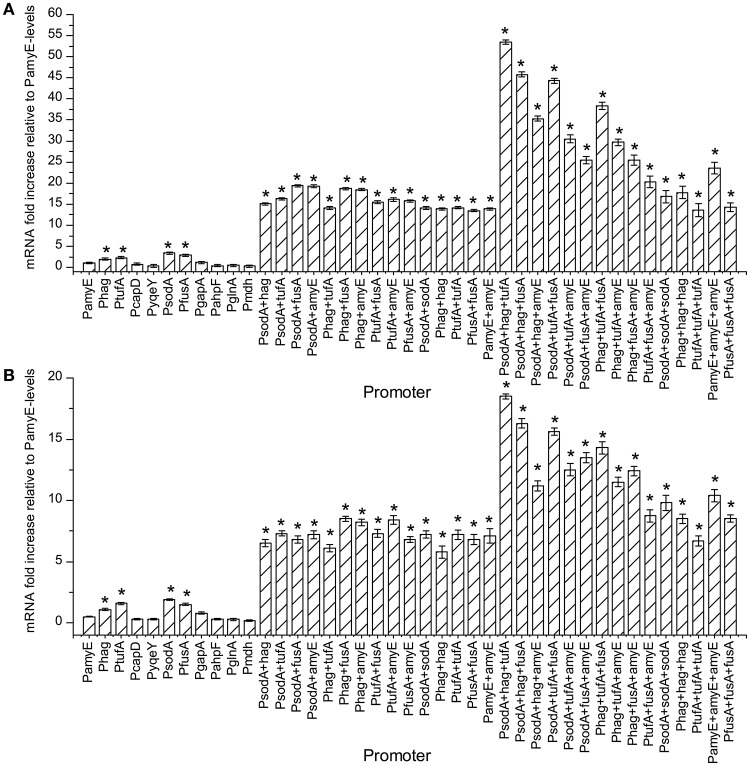
The effect of different promoters and their combinations on the amount of mRNA. Normalized gene expression (ΔΔCT) reported. The reference gene is the 16S ribosomal gene, PamyE is control, graphed relative to zero. Panel **(A)** is the sample cultured for 24 h. Panel **(B)** is the sample cultured for 48 h. All tests repeated three time. “*”means the amount of mRNA increased significantly (*P* < 0.05) compared to control promoter PamyE at 24 or 48 h, respectively.

**Figure 4:**
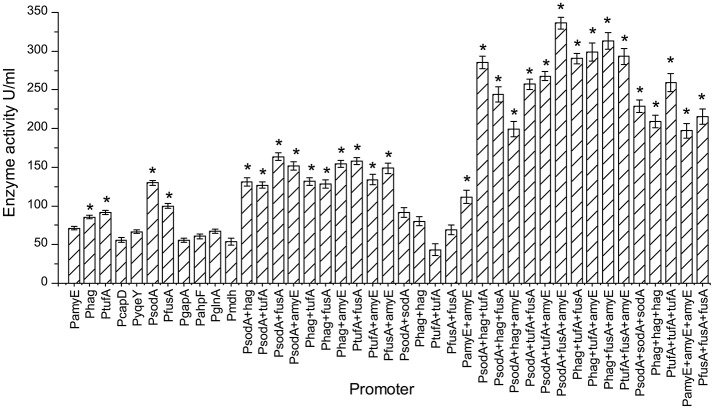
The pullulanase activity of all engineering strains. The effect of different promoters and their combinations on the activity of pullulanase. “*”means the activity of pullulanase increased significantly (*P* < 0.05) compared to control promoter PamyE.

The amount of mRNA at 24 and 48 h of t strains were calculated by qPCR, and the result are shown in Figure 3. As the number of promoter's increases, the amount of mRNA increased continuously. The mRNA levels of the strains containing Phag, PtufA, PsodA, and PfusA were higher than the strain containing PamyE. Whereas the strains with dual promoters were higher the level in strains with single promoters, which were 15.5–19.2 times higher than that of the strain containing PamyE at 24 h and 11.6–17 times higher than that at 48 h, respectively (Figure 4). The strain containing PsodA+hag+tufA had the highest level of pullulanase mRNA, which was 53.5 and 37 times higher than that in the strain containing PamyE at 24 and 48 h, respectively. However, the expressed enzyme activity (285 U/ml) was lower than that in the strain containing PsodA+fusA+amyE (336 U/ml). It suggested that the enzyme activity would be not only related to the strength of the promoter, but also related to other factors.

### The effect of different promoters and their combinations on the activity of pullulanase

The expression of pullulanase from a single, dual, or triple promoter is shown in Figure 4. Recombinant strains were selected and cultured for 48 h in CSA medium at 37°C for 48 h. The pullulanase activity of strain containing triple promoter was higher than that in the strain containing single or dual promoter. Among the single promoter isolates, the enzyme activity obtained from the strain with PsodA was the highest (129.8 U/mL), which was 1.82 times higher than that of control strain with PamyE (71.1 U/ml). And the enzyme activity levels in the strains containing PsodA+fusA, PtufA+fusA, Phag+amyE, or PsodA+amyE were more than 150 U/mL, which was more than 2 times higher than that of the control strain with PamyE. Among the triple-promoter strains, the pullulanase activity in the strain containing PsodA+fusA+amyE was 336 U/mL, which is 4.72 times higher than that of the strain with PamyE (Figure 4).The results indicated that pullulanase activity was significantly improved by the multiple promoter combinations.

**Figure 5:**
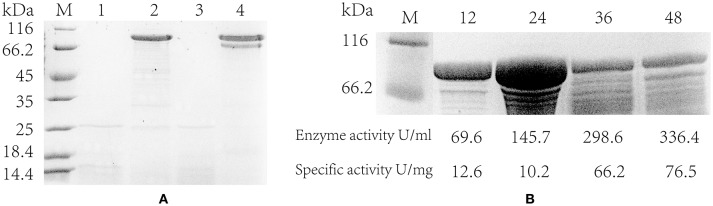
The extracellular protein and specific activity of engineered strain. **(A)** Detecting the expression of pullulanase in flasks by SDS-PAGE. lane 1 and 3 are the blank control (host strain without *pulA* gene); lane 2 and 4 are the engineering strain with PamyE; lane 1–2 are the extracellular protein in the supernatant at 24 h; lane 3–4 are the extracellular protein at 48 h. **(B)** The SDS-PAGE of engineering strains with PsodA+fusA+amyE in flasks, lane 1 is protein marker, lane 2–5 are the extracellular protein in the supernatant at 12, 24, 36, and 48 h. Below the figure is the data of enzyme activity and specific activity.

In addition, the pullulanase proteins expressed in the strain containing PsodA+fusA+amyE and the blank strain was confirmed by SDS-PAGE electrophoresis. It suggested that the pullulanase expressed successfully and a band was clearly shown in the engineered strains at 24 and 48 h, whereas the blank strain did not appeared the target band (Figure 5A). However, it is noteworthy that the pullulanase protein yield of the strain with PsodA+fusA+amyE reached a maximum, 14.3 g/L at 24 h, whereas its enzyme activity and specific enzyme activity (145.7 U/mL and 10.2 U/mg) was lower than those at 36 h (298.7 U/mL and 66.2 U/mg) and 48 h (336.4 U/mL and 76.5 U/mg), respectively (Figure 5B). This may be that a portion of pullulanase was misfolded or not modified at 24 h, and then the misfolded enzyme was likely degraded and was modified, thus, the specific enzyme activity was increased at 36 and 48 h.

### The pullulanase yield from engineered strains in flasks and 50-L fermenters

**Figure 6:**
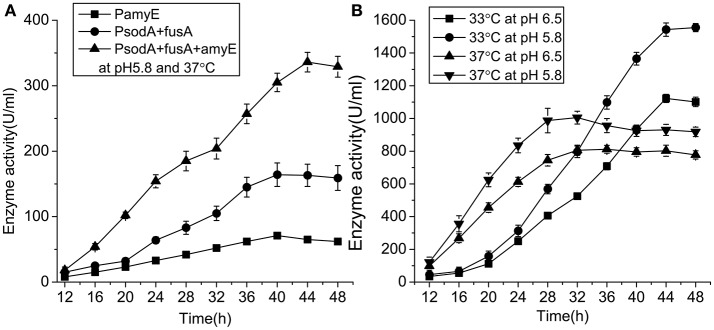
The enzyme activity of engineered strains. **(A)** The enzyme activity of engineered strains with different promoter in 250 ml flasks at pH 5.8 and 37°C. **(B)** The enzyme activity of engineered strain with the promoter PsodA+fusA+amyE in 50-Liter fermenter under different conditions.

The activity of pullulanase in the strain with PsodA+fusA+amyE over 48 h in shake flasks was increased from 71 to 336.4 U/mL following optimization of the growth conditions (Figure 6A). The strains with PamyE, PsodA+fusA, and PsodA+fusA+amyE were cultured in 250 mL flasks containing 50 mL of medium. The results of the enzyme activity assay indicated that the enzyme was produced starting at ~12, and reached a maximum at 40–44. The strain with PsodA+fusA+amyE was subsequently cultured in a 50-L fermenter at either 37 or 33°C, and the pH was maintained at either 5.8 or 6.5. At pH 5.8, enzyme activity was higher at 33°C (1,555 U/mL) than at 37°C (1,005 U/mL). In addition, enzyme activity was higher at pH 5.8 (1,555 U/mL) than at pH 6.5 (1,122 U/mL) at 33°C (Figure 6B).

## Discussion

We analyzed three transcriptome data sets from *B. subtilis* to select the genes with the highest expression levels. The promoters of the top 10 genes were determined through predictive bioinformatic analyses and were used to express pullulanase in *B. subtilis*. Four promoters (PsodA, Phag, PtufA, and PfusA) were stronger than PamyE. Transcriptome mining, with the goal of engineering promoter-induced modifications to increase gene expression, has been previously reported. For example, Liu et al. (2017) selected a promoter, PBL9, from 3,595 genes and 1,249 operons in a *B. licheniformis* transcriptome, that was 23% stronger than P43. Liao et al. (2015) screened the candidate promoter Pr2 (the promoter of *sigW*) from 288 genes with higher expression levels (RPKM values) than the control gene P43, and observed the strongest β-galactosidase activity in post-log phase. Park et al. (2007) identified a cadmium-inducible promoter via transcriptome analysis of *Hansenula polymorpha* SEO1 that had broad specificity for heavy metals and was also responsive to arsenic and mercury. This study of pullulanase expression in *B. subtilis* reinforces the idea that selecting promoters from transcriptome data is a good approach for identifying strong promoters and can be used to optimize the expression of industrially important microbial products, saving time, reducing costs, and improving safety. This study is also the first to select promoters based on *B. subtilis* transcriptome data, showing that it is a viable option to modify expression of pullulanase.

The yield of pullulanase from a single promoter was unsatisfactory because of insufficient strength. Some researchers proved that optimizing the −35 and −10 regions of promoter could enhance promoter strength. Jiao et al. (2017) constructed a super-strong promoter, Pg3, by −35 and −10 regions mutations, which was 1.63 times higher than that before mutation in *B. subtilis*. In addition, Feng et al. (2017) generated P43 promoter variants, which was 1.77 times higher than P43 promoter. Research has also shown that artificial dual-promoters are typically stronger than single promoters. For example, the dual promoter PgsiB-PhpaII was shown to be stronger than PhpaII, PyxiE, P43, PgsiB, Pluxs, or PaprE alone (Guan C. et al., 2016). In addition, the strength of the dual promoters PhpaII-PamyR and PhpaII-Pblma was 11- to 12-fold higher than the single promoter PhpaII in *B. subtilis* (Kang et al., 2010). Sinah et al. (2012) also constructed a set of two promoters for high protein expression in both *E. coli* and *S. cerevisiae*. Therefore, we combined strong single promoters to generate artificial multiple-promoter systems to increase the yield of the target protein. Pullulanase mRNA transcript levels and enzyme activity were significantly increased with the number of promoters (Figure 5).

PsodA+fusA+amyE was a semi-constitutive promoter constructed from PsodA and PfusA, which are constitutive promoters, and PamyE, which is a starch- and maltose-inducible promoter. Therefore, this triple promoter system could be induced in CSA medium and did not require an inducer. Constitutive promoters are advantageous in large-scale industrial production because they do not require an inducer. This simplifies the composition of the medium and the fermentation conditions, thus reducing production costs. Although the activity of pullulanase heterologously expressed under various conditions was as high as 580 U/mL (Nie et al., 2013) and 2523.5 U/mL (Zou et al., 2014) in *E. coli*, it was comparatively low in *B. subtilis*, at 5.7 U/mL (Chen et al., 2001), 2.82 U/mL (Wang et al., 2014), and 24.5 U/mL (Song et al., 2016). In this study, the yield of pullulanase was as high as 1,555 U/mL, which is the highest yield reported to date. In addition, we recently improved pullulanase activity to 2,180 U/mL by optimizing the medium composition and controlling the fermentation conditions.

Interestingly, the strain containing PsodA+hag+tufA had the highest mRNA expression, but not the highest enzyme activity (Figures 3, 5). In fact, the enzyme activity in the strain containing PsodA+hag+tufA was only 84.8% of that in the strain containing PdosA+fusA+amyE (Figure 3). This suggests that post-transcriptional modifications may modulate enzyme levels or activity. Alternatively, the overexpressed mRNA might not be used as a template for translation due to limited amounts of tRNA or ribosomes (Yuan and Wong, 1995; Wu et al., 1998). Further, the concentration of pullulanase protein was 14.3 g/L at 24 h, while its specific activity was only 10.2 U/mg. This indicates that some of the pullulanase might be misfolded and have no enzymatic activity (Li et al., 2004; Yan and Wu, 2017). In the future studies, we would like to determine the structure of pullulanase by NMR or X-ray crystallography to confirm protein misfolding and explore how changes in the culture conditions or chaperones can be employed to improve the folding rate.

In this study, four strong promoters from *B. subtilis* were identified by analyzing transcriptome data in GenBank, and these promoters were used to express pullulanase in *B. subtilis* BS001. Both gene expression and protein production increased significantly with increasing tandem combinations of promoters. The enzyme activity of the strain with the triple promoter complex PsodA+fusA+amyE reached 336.4 U/mL in a shake flask and 1,555 U/mL in a 50-L fermenter, which was 4.73 times higher than that of the strain with PamyE. The strain with PsodA+hag+tufA showed the highest mRNA levels, which were 53.5 and 37 times higher than that of the strain with PamyE at 24 and 48 h, respectively. Taken together, these results demonstrate that bioinformatic analysis in combination with genetic recombination technology can be used to develop microbial bioproduct advancements that can quickly and safely benefit industrial production in a cost-effective way. We will furtherly upgrade the expression strength by promoter mutation and optimize fermentation conditions in order to furtherly enhance the pullulanase production.

## Availability of data and materials

The datasets used and/or analyzed during the current study are available from the corresponding authors on reasonable request.

## Author contributions

FM designed and performed the experiments, analyzed the data, and wrote the manuscript. XZ and TN constructed plasmids and transformed into host strain *B.subtilis* B001. FL and XB conceived the project, designed the experiments. YL and FT wrote a part of the Discussion section and helped with language editing. ZL designed the research content and analyzed the data. All authors read and approved the final manuscript.

### Conflict of interest statement

The authors declare that the research was conducted in the absence of any commercial or financial relationships that could be construed as a potential conflict of interest.

## Abbreviations

P: promoter
PamyE: promoter amyE
RPKM: Reads Per Kilo-bases per Million-reads
STAB-SD: the Shine-Dalgarno mRNA stabilizing sequence.
