# Supplementary material for: Enhanced Expression of Pullulanase in Bacillus subtilis by New Strong Promoters Mined From Transcriptome Data, Both Alone and in Combination
Source: Front Microbiol. 2018 Nov 2;9:2635. doi: 10.3389/fmicb.2018.02635 (PMC6224515; doi:10.3389/fmicb.2018.02635)
Supplement: Supplementary file 1 [file Data_Sheet_1.pdf]

Enhanced expression of pullulanase in *Bacillus subtilis* by new strong promoters mined from transcriptome data, both alone and in combination

Fanqiang Meng[1], Xiaoyu Zhu[1], Ting Nie[1], Fengxia Lu[1], Xiaomei Bie[1], Yingjian Lu[2], Frances Trouth[3], Zhaoxin Lu[1*]

[1] College of Food Science and Technology, Nanjing Agricultural University, 1 Weigang, Nanjing, China, 210095.
[2] Department of Food Science and Nutrition, University of Maryland, College Park, Maryland, USA, 20742.
[3] Department of Plant Science and Landscape Architecture, University of Maryland, College Park, Maryland, USA, 20742.
Fanqiang Meng: 2016208025@njau.edu.cn
Xiaoyu Zhu: zhuxiaoyu@njau.edu.cn
Ting Nie: 2016208007@njau.edu.cn
Fengxia Lu: lufengxia@njau.edu.cn
Xiaomei Bie: bxm43@njau.edu.cn
Yingjian Lu: yjlu@umd.edu
Frances Trouth: Frances.Trouth@ars.usda.gov
*** Correspondence:**
Corresponding Author: Zhaoxin Lu, Ph.D., College of Food Science and Technology, Nanjing Agriculture University, 1 Weigang, Nanjing 210095, China
Email: fmb@njau.edu.cn Tel. (Fax): 0086-25-84396583

Table S1 primers used in this study

Name	Description	Primers	Restriction sites
F1	amplify *amyE*-up arm	ggatccGACTCCGAAGTAAGTCTTC	*Bam*HI
R1	amplify *amyE*-up arm	cgtcgacgCGCCCGATCAGACCAGTTTTTAA	*SgrD*I
F2	amplify *amyE*-down arm	ccatggCAAGTGAACGATGGTAAACT	*Nco*I
R2	amplify *amyE*-down arm	agatctTCAATGGGGAAGAGAACCG	*Bgl*II
F3	amplify mRNA stab	gaattcAGGGATGCCTAAAAACGAAG	*Eco*RI
R3	amplify mRNA stab	acgcgtCACTTTTCATAATACATAAT	*Mlu*I
F4	amplify *pulA*	gtcgacGCAGCAGCTAAACCCGCTG	*Sal*I
R4	amplify *pulA*	cgtcgacgTTAACTTTTTACCGTGGT	*Sgr*DI
F5	amplify signal peptide *amyE*	acgcgtATGTTTGCAAAACGATTCA	*Mlu*I
R5	amplify signal peptide *amyE*	gtcgagAGCACTCGCAGCCGCCGGT	*Sal*I
F6	amplify promoter amyE to form pCBS4	cccgggTAGAGTGATTGTGATAAT	*Sma*I
R6	amplify promoter amyE to form pCBS4	gaattcTCTTGACACTCCTTCTTTG	*Eco*RI
F7	amplify promoter hag to form pCBS5	cccgggGGATTTTTTTATTTTTGTAT	*Sma*I
R7	amplify promoter hag to form pCBS5	gaattcTGTTTTGTTCCTCCCTGAAT	*Eco*RI
F8	amplify promoter tufA to form pCBS6	cccgggTTGATTTTGCCGCTTAACTC	*Sma*I
R8	amplify promoter tufA to form pCBS6	gaattcTCTAAAATCCTCCTTAAG	*Eco*RI
F9	amplify promoter cspD to form pCBS7	cccgggGGCGTACTTGAGAGCATACGA	*Sma*I
R9	amplify promoter cspD to form pCBS7	gaattcATTGCTTAATTCCTCCTAG	*Eco*RI
F10	amplify promoter yqeY to form pCBS8	cccgggAACGAATCGCTTGAAGATGCTC	*Sma*I
R10	amplify promoter yqeY to form pCBS8	gaattcAAATCCACCCTCTTTTAGAATTTGC	*EcoR*I
F11	amplify promoter sodA to form pCBS9	cccgggGAAATGCTGGCGGCAGGTTTAATG	*Sma*I
R11	amplify promoter sodA to form pCBS9	gaattcGATAATTCCTCCTTAGTATAT	*Eco*RI
F12	amplify promoter fusA to form pCBS10	cccgggCTGGTGCTGCTGTTAAGAAAC	*Sma*I
R12	amplify promoter fusA to form pCBS10	gaattcTGGGTAATTTCTCCTTCCT	*Eco*RI
F13	amplify promoter gapA to form pCBS11	cccgggATCGAGGCTTACTTTAAAAAGC	*Sma*I
R13	amplify promoter gapA to form pCBS11	gaattcGATTGTTTCCTCCTTTAAAT	*Eco*RI
F14	amplify promoter ahpFto form pCBS12	cccgggTTGACAAAAAATATATATTAATT	*Sma*I
R14	amplify promoter ahpF to form pCBS12	gaattcAATGTATATTCCTCCTAAAAATG	*Eco*RI
F15	amplify promoter glnA to form pCBS13	cccgggATTTTTTAAAAATTTCTCTGGATTTG	*Sma*I
R15	amplify promoter glnA to form pCBS13	gaattcCTCAATTTCCTCCTTTTCTTAAC	*Eco*RI
F16	amplify promoter mdh to form pCBS14	cccgggGACTGAAGTGAAATGTTC	*Sma*I
R16	amplify promoter mdh to form pCBS14	gaattcGTCTCTCTTCTCCTTTATG	*Eco*RI
F17	amplify sodA promoter to form pCBS15,16,17,18,27，40	ggtaccGAAATGCTGGCGGCAGGTTTAATG	*Kpn*I
R17	amplify sodA promoter to form pCBS15,16,17,18,27，40	cccgggGATAATTCCTCCTTAGTATAT	*Sma*I
F18	amplify hag promoter to form pCBS19,20,21,25,30,31,32,41	ggtaccGGATTTTTTTATTTTTGTAT	*Kpn*I
R18	amplify hag promoter to form pCBS19,20,21,25,30,31,32,41	cccgggTGTTTTGTTCCTCCCTGAAT	*Sma*I
F19	amplify tufA promoter to form pCBS22,23,26,33,34,36,37,42	ggtaccTTGATTTTGCCGCTTAACTC	*Kpn*I

R19	amplify tufA promoter to form pCBS22,23,26,33,34,36,37,42	cccgggTCTAAAATCCTCCTTAAG	*Sma*I
F20	amplify fusA promoter to form pCBS24,28,35,38,39	ggtaccCTGGTGCTGCTGTTAAGAAAC	*Kpn*I
R20	amplify fusA promoter to form pCBS24,28,35,38,39	cccgggTGGGTAATTTCTCCTTCCT	*Sma*I
F21	amplify amyE promoter to form pCBS29,43	ggtaccTAGAGTGATTGTGATAAT	*Kpn*I
R21	amplify amyE promoter to form pCBS29,43	cccgggTCTTGACACTCCTTCTTTG	*Sma*I
F22	amplify sodA promoter to form pCBS30-35,40	ccatggGAAATGCTGGCGGCAGGTTTAAT	*Nco*I
R22	amplify sodA promoter to form pCBS30-35,40	ggtaccGATAATTCCTCCTTAGTATAT	*Kpn*I
F23	amplify hag promoter to form pCBS36-38,41	ccatggGGATTTTTTTATTTTTGTAT	*Nco*I
R23	amplify hag promoter to form pCBS36-38,41	ggtaccTGTTTTGTTCCTCCCTGAAT	*Kpn*I
F24	amplify tufA promoter to form pCBS39,42	ccatggTTGATTTTGCCGCTTAACTC	*Nco*I
R24	amplify tufA promoter to form pCBS39,42	ggtaccTCTAAAATCCTCCTTAAG	*Kpn*I
F25	amplify amyE promoter to form pCBS43	ccatggTAGAGTGATTGTGATAAT	*Nco*I
R25	amplify amyE promoter to form pCBS43	ggtaccTCTTGACACTCCTTCTTTG	*Kpn*I

Lowercase letters are restriction site

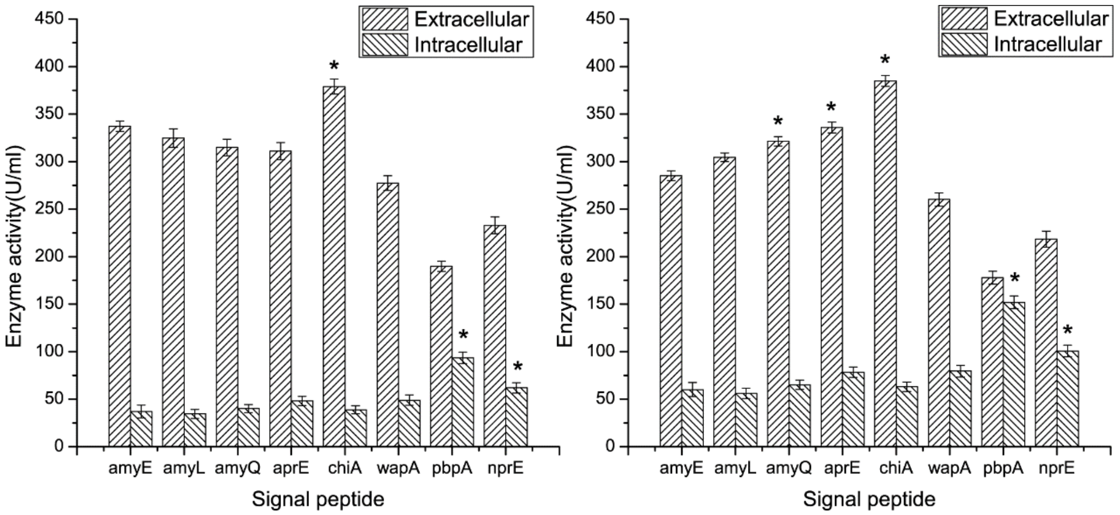

Figure S1 the effect of signal peptide on the extracellular and intracellular enzyme activity.

(a) the extracellular and intracellular enzyme activity of engineering strain PsodA+fusA+amyE with the different signal peptide. (b) the extracellular and intracellular enzyme activity of engineering strain PsodA+hag+tufA with the different signal peptide. "*"means the effect of the increase was significant (P<0.05) compared to control group PamyE.

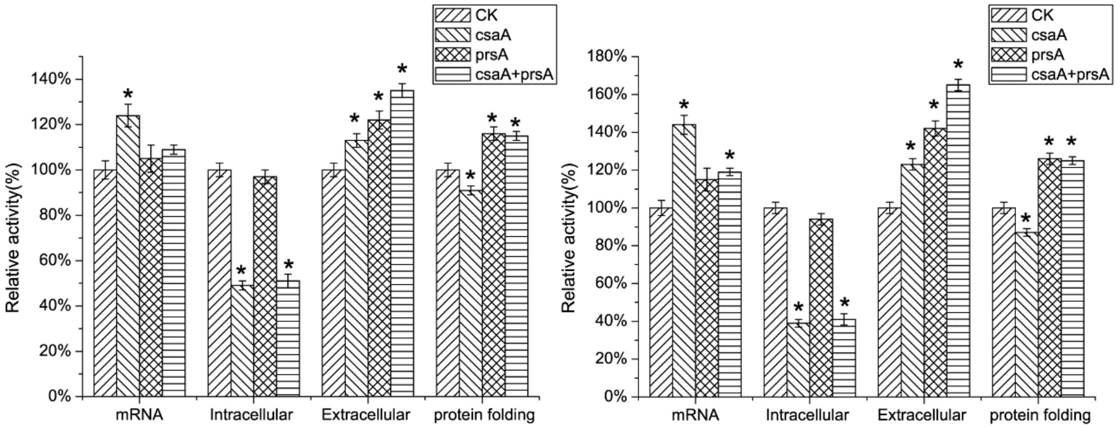

(a) (b)

Figure S2 the effect of chaperone on the secretion efficiency and protein folding rate.

(a) The effect of chaperone on the amount of mRNA, intercellular and extracellular enzyme activity, and extracellular protein folding rate of strain PsodA+fusA+amyE. (b) The effect of chaperone on the amount of mRNA, intercellular and extracellular enzyme activity, and extracellular protein folding rate of strain PsodA+hag+tufA. "*"means the difference was significant (P<0.05) compared to control group PamyE.

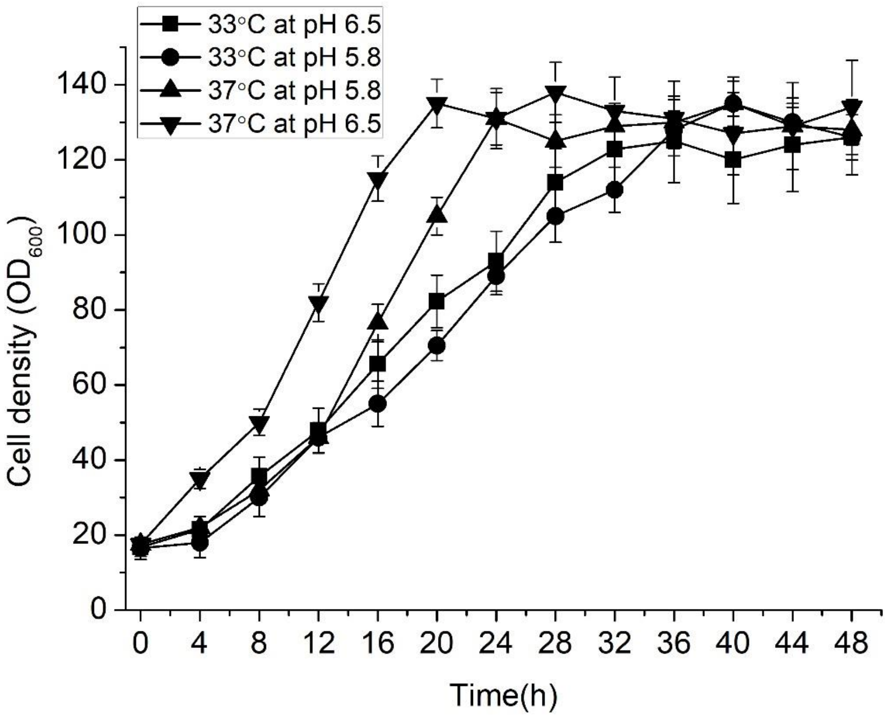

Figure S3 The cell density (OD_{600}) of engineered strains with the promoter PsodA+fusA+amyE in 50-Liter fermenter under different conditions.

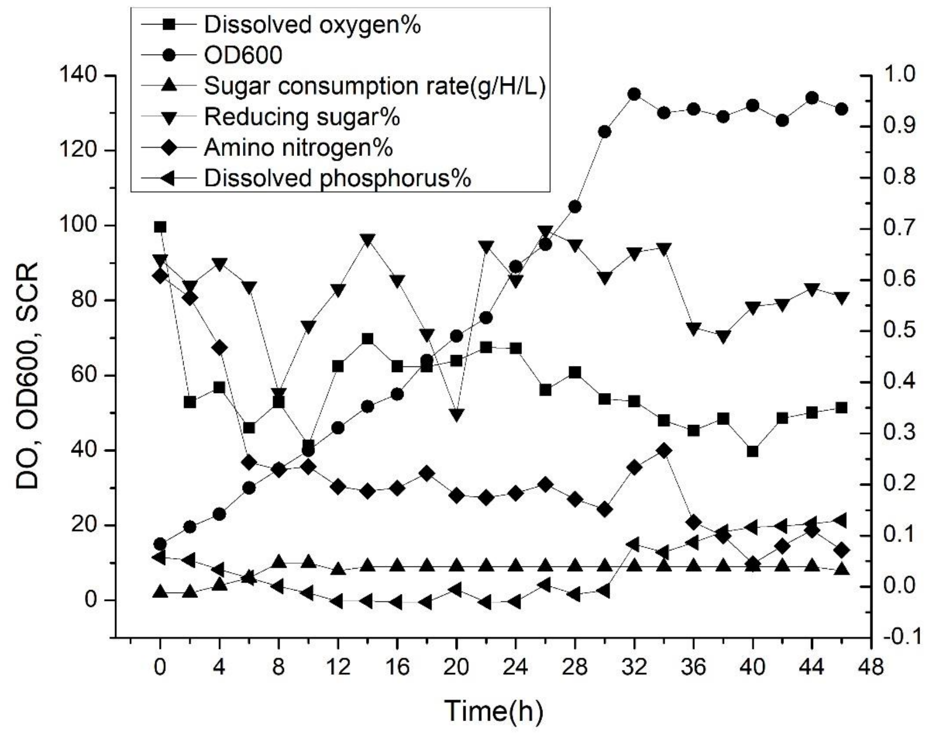

Figure S4 The fermentation related parameters of engineered strains with the promoter PsodA+fusA+amyE in 50-

Liter fermenter. DO is dissolved oxygen, OD600 is the cell density, SCR is sugar consumption rate, RS is reducing sugar in supernatant, AN is free form amino nitrogen, DP is dissolved phosphorus.

The sequence of homology arm, pulA and promoters.

amyE-up homology arm

AGAATGAAGTAAGAGGGATTTTTGACTCCGAAGTAAGTCTTCAAAAAATCAAATAAGGAGTGTC
AAGAATGTTTGCAAAACGATTCAAAACCTCTTTACTGCCGTTATTCGCTGGATTTTTATTGCTGTT
TCATTTGGTTCTGGCAGGACCGGCGGCTGCGAGTGCTGAAACGGCGAACAAATCGAATGAGCT
TACAGCACCGTCGATCAAAAGCGGAACCATTCTTCATGCATGGAATTGGTCGTTCAATACGTTA
AAACACAATATGAAGGATATTCATGATGCAGGATATACAGCCATTCAGACATCTCCGATTAACC
AAGTAAAGGAAGGGAATCAAGGAGATAAAAGCATGTCGAACTGGTACTGGCTGTATCAGCCGA
CATCGTATCAAATTGGCAACCGTTACTTAGGTACTGAACAAGAATTTAAAGAAATGTGTGCAGC
CGCTGAAGAATATGGCATAAAGGTCATTGTTGACGCGGTCATCAATCATACCACCAGTGATTAT
GCCGCGATTTCCAATGAGGTTAAGAGTATTCCAAACTGGACACATGGAAACACACAAATTAAAA
ACTGGTCTGATCGGGCG

amyE-down homology arm

CAAGTGAACGATGGTAAACTGACAGGCACGATCAATGCCAGGTCTGTAGCTGTGCTTTATCCTG
ATGATATTGCAAAAGCGCCTCATGTTTTCCTTGAGAATTACAAAACAGGTGTAACACATTCTTTC
AATGATCAACTGACGATTACCTTGCGTGCAGATGCGAATACAACAAAAGCCGTTTATCAAATCA
ATAATGGACCAGAGACGGCGTTTAAGGATGGAGATCAATTCACAATCGGAAAAGGAGATCCAT
TTGGCAAAACATACACCATCATGTTAAAAGGAACGAACAGTGATGGTGTAACGAGGACCGAGA
AATACAGTTTTGTTAAAAGAGATCCAGCGTCGGCCAAAACCATCGGCTATCAAAATCCGAATCA
TTGGAGCCAGGTAAATGCTTATATCTATAAACATGATGGGAGCCGAGTAATTGAATTGACCGGA
TCTTGGCCTGGAAAACCAATGACTAAAAATGCAGACGGAATTTACACGCTGACGCTGCCTGCG
GACACGGATACAACCAACGCAAAAGTGATTTTTAATAATGGCAGCGCCCAAGTGCCCGGTCAG
AATCAGCCTGGCTTTGATTACGTGCTAAATGGTTTATATAATGACTCGGGCTTAAGCGGTTCTCT
TCCCCATTGA

Sequence of pulA

GCAGCAGCTAAACCCGCTGTAAGCAACGCTTATTTAGATGCTTCAAACCAGGTGCTGGTTAAAC
TTAGCCAGCCGTTAACTCTTGGGGAAGGCGCAAGCGGCTTTACGGTTCATGACGACACAGCAA
ATAAGGATATTCCAGTGACATCTGTGAAGGATGCAAGTCTTGGTCAAGATGTAACCGCTGTTTTG
GCAGGTACCTTCCAACATATTTTTGGAGGTTCCGATTGGGCACCTGATAATCACAGTACTTTATT
AAAAAAGGTGACTAACAATCTCTATCAATTCTCAGGAGATCTTCCTGAAGGAAACTACCAATAT
AAAGTGGCTTTAAATGATAGCTGGAATAATCCGAGTTACCCATCTGACAACATTAATTTAACAGT
CCCTGCCGGCGGTGCACACGTCACTTTTTCGTATATTCCGTCCACTCATGCAGTCTATGACACAA
TTAATAATCCTAATGCGGATTTACAAGTAGAAAGCGGGGTTAAAACGGATCTCGTGACGGTTAC
TCTAGGGGAAGATCCAGATGTGAGCCATACTCTGTCCATTCAAACAGATGGCTATCAGGCAAAG
CAGGTGATACCTCGTAATGTGCTTAATTCATCACAGTACTACTATTCAGGAGATGATCTTGGGAA
TACCTATACACAGAAAGCAACAACCTTTAAAGTCTGGGCACCAACTTCTACTCAAGTAAATGTTC

TTCTTTATGACAGTGCAACGGGTTCTGTAACAAAAATCGTACCTATGACGGCATCGGGCCATGG
TGTGTGGGAAGCAACGGTTAATCAAAACCTTGAAAATTGGTATTACATGTATGAGGTAACAGGC
CAAGGCTCTACCCGAACGGCTGTTGATCCTTATGCAACTGCGATTGCACCAAATGGAACGAGAG
GCATGATTGTGGACCTGGCTAAAACAGATCCTGCTGGCTGGAACAGTGATAAACATATTACGCC
AAAGAATATAGAAGATGAGGTCATCTATGAAATGGATGTCCGTGACTTTTCCATTGACCCTAATT
CGGGTATGAAAAATAAAGGGAAGTATTTGGCTCTTACAGAAAAAGGAACAAAGGGCCCTGACA
ACGTAAAGACGGGGATAGATTCCTTAAAACAACTTGGGATTACTCATGTTCAGCTTATGCCTGTT
TTCGCATCTAACAGTGTCGATGAAACTGATCCAACCCAAGATAATTGGGGTTATGACCCTCGCA
ACTATGATGTTCCTGAAGGGCAGTATGCTACAAATGCGAATGGTAATGCTCGTATAAAAGAGTT
TAAGGAAATGGTTCTTTCACTCCATCGTGAACACATTGGGGTTAACATGGATGTTGTCTATAATC
ATACCTTTGCCACGCAAATCTCTGACTTCGATAAAATTGTACCAGAATATTATTACCGTACGGAT
GATGCAGGTAATTATACCAACGGATCAGGTACTGGAAATGAAATTGCAGCCGAAAGGCCAATG
GTTCAAAAATTTATTATTGATTCCCTTAAGTATTGGGTCAATGAGTATCATATTGACGGCTTCCGT
TTTGACTTAATGGCGCTGCTTGGAAAAGACACGATGTCCAAAGCTGCCTCGGAGCTTCATGCTA
TTAATCCAGGAATTGCACTTTACGGTGAGCCATGGACGGGTGGAACCTCTGCACTGCCAGATGA
TCAGCTTCTGACAAAAGGAGCTCAAAAAGGCATGGGAGTAGCGGTGTTTAATGACAATTTACGA
AACGCGTTGGACGGCAATGTCTTTGATTCTTCCGCTCAAGGTTTTGCGACAGGTGCAACAGGCT
TAACTGATGCAATTAAGAATGGCGTTGAGGGGAGTATTAATGACTTTACCTCTTCACCAGGTGA
GACAATTAACTATGTCACAAGTCATGATAACTACACCCTTTGGGACAAAATAGCCCTAAGCAAT
CCTAATGATTCCGAAGCGGATCGGATTAAAATGGATGAACTCGCACAAGCAGTTGTTATGACCT
CACAAGGCGTTCCATTCATGCAAGGCGGGGAAGAAATGCTTCGTACAAAAGGCGGCAACGACA
ATAGTTATAATGCAGGCGATGCGGTCAATGAGTTTGATTGGAGCAGGAAAGCTCAATATCCAGA
TGTTTTCAACTATTATAGCGGGCTAATCCACCTTCGTCTTGATCACCCAGCCTTCCGCATGACGA
CAGCTAATGAAATCAATAGCCACCTCCAATTCCTAAATAGTCCAGAGAACACAGTGGCCTATGA
ATTAACTGATCATGTTAATAAAGACAAATGGGGAAATATCATTGTTGTTTATAACCCAAATAAAA
CTGTAGCAACCATCAATTTGCCGAGCGGGAAATGGGCAATCAATGCTACGAGCGGTAAGGTAG
GAGAATCCACCCTTGGTCAAGCAGAGGGAAGTGTCCAAGTACCAGGTATATCTATGATGATCCT
TCATCAAGAGGTAAGCCCAGACCACGGTAAAAAGTAA

mRNA stable sequence
AGGGATGCCTAAAAACGAAGAACATTAAAAACATATATTTGCACCGTCTAATGGATTTATGAAA
AATCATTTTATCAGTTTGAAAATTATGTATTATGAAAAGTG

.
The sequence of PamyE
TAGAGTGATTGTGATAATTTTAAATGTAAGCGTTAACAAAATTCTCCAGTCTTCACATCGGTTTGA
AAGGAGGAAGCGGAAGAATGAAGTAAGAGGGATTTTTGACTCCGAAGTAAGTCTTCAAAAAAT
CAAATAAGGAGTGTCAAGA

The sequence of Phag
GGATTTTTTTATTTTTGTATTAACAAAATCAGAGACAATCCGATATTAATGATGTAGCCGGGAGG
AGGCGCAAAAGACTCAGCCAGTTACAAAATAAGGGCACAAGGACGTGCCTTAACAACATATTC
AGGGAGGAACAAAACA

The sequence of PtufA
TTGATTTTGCCGCTTAACTCAAGTATAACTACTATTGTAAGATGAGGAAGTGAAAGCTTTCTTTCA
CTTCCTATCACTCTATACATTACTAATTAAAAGCTCTTAAGGAGGATTTTAGA
The sequence of PcspD
GGCGTACTTGAGAGCATACGAAAATCGTGTGCTCTTTTTATTTATATTCAGCCATCAATAAAAGC
GGTTACATTTTTTTATGGAACTTGCCCTTCTTTTGAAAAATAAGCCGTTTCGCAACTTGACGGGTG
CTCCCAGATGGTGTATAGTTGAACCATCATTTAACAATGAATCAAAGTTAGATGATGACAAAATT
TTTTTTAGCACATGGGTGCTACAGTACTAGGAGGAATTAAGCAAT
The sequence of PyqeY
AACGAATCGCTTGAAGATGCTCTTCGTCGCTTCAAACGCAGTGTATCAAAGACAGGTACTTTGC
AAGAAGCAAGAAAGCGTGAATTTTATGAAAAACCTAGCGTAAAGCGCAAGAAAAAGTCTGAAG
CTGCTAGAAAACGCAAATTCTAAAAGAGGGTGGATTT
The sequence of PsodA
GAAATGCTGGCGGCAGGTTTAATGGGGCAGATTATCGGTTAAAGTGAAATATGTCCGCTGAAAT
GAGCCAAATCGTGAGCTTTTTCATTGATTACAAAGCATTCTTTTGTTACATTGAATGAAGAAAGG
CTCGCTGTGAAGCGGCATTTCAGTACATATATACTAAGGAGGAATTATC
The sequence of PfusA
CTGGTGCTGCTGTTAAGAAACGTGAAGACACTCATAAGATGGCTGAAGCAAACAAAGCATTTGC
TCACTATCGCTGGTAGGATTTATAGATTAACAAAACACAATTTCCTAATAAGGAAGGAGAAATTA
CCCA

The sequence of PgapA
ATCGAGGCTTACTTTAAAAAGCCACGCAACACGGTTCTCGTCACAGACGAAGGAGCCGCAAAG
AAGTTATTAAGGGATGAATAATCCCTCAATATAAATATCTCTCACTTATTTAAAGGAGGAAACAA
TC
The sequence of PahpF
TTGACAAAAAATATATATTAATTAATAATTCATATATAATTAGAATTATTATTGAAAGCGATTATG
CTTTCTAATACATTTTTAGGAGGAATATACATT
mdh

The sequence of PglnA
ATTTTTTAAAAATTTCTCTGGATTTGATGTTAAGAATCCTTACATCGTATTGACACATAATATAAC
ATCACCTATAATGAAACTAAGTTAAGAAAAGGAGGAAATTGAG

The sequence of Pmdh
GACTGAAGTGAAATGTTCAGAGTTCGGAGAAGAACTGATCAAAAACATGGACTAAGCAAGGAA
AAAGCCTAAAACTAGCCATAAAGGAGAAGAGAGAC

SPamyE
ATGTTTGCAAAACGATTCAAAACCTCTTTACTGCCGTTATTCGCTGGATTTTTATTGCTGTTTCATT
TGGTTCTGGCAGGACCGGCGGCTGCGAGTGCT

SPamyL
ATGATTCAAAAACGAAAGCGTGCCATATCATGGGGTCTGGTGTTTTTCTTAACACTACTGTTTGT

CACGCTGCCGGTATCTAAAACTTGGGCTGCA

SPamyQ
ATGATTCAAAAACGAAAGCGGACAGTTTCGTTCAGACTTGTGCTTATGTGCACGCTCTTATTTGT
CAGTTTGCCGATTACAAAAACATCAGCTGTA

SPaprE
GTGAGAAGCAAAAAATTGTGGATCAGCTTGTTGTTTGCGTTAACGTTAATCTTTACGATGGCGTT
CAGCAACATGTCTGCGCAG

SPchiA
ATGTTGATCAACAAAAGCAAAAAGTTTTTCGTTTTTTCTTTCATTTTTGTTATGATGCTGAGCCTCT
CATTTGTGAATGGGGAAGTTGCAAAAGCC

SPwapA
ATGAAAAAAAGAAAGAGGCGAAACTTTAAAAGGTTCATTGCAGCATTTTTAGTGTTGGCTTTAAT
GATTTCATTAGTGCCAGCCGATGTACTAGCAAAATCTA

SPpbpA
ATGAGGAGAAATAAACCAAAAAAGCAAAATCATAAAGAGAAAAAGAAGTCGCTTCCGATCCGG
CTGAATATTTTATTTTTAGCTGCCTTTGTTATATTTACCTG

SPnprE
GTGGGTTTAGGTAAGAAATTGTCTGTTGCTGTCGCTGCTTCGTTTATGAGTTTATCAATCAGCCTG
CCAGGTGTTCAGGCT
